# Cultural and social factors associated with generalized anxiety disorder among adolescent mothers during the postpartum period in Malawi: a cross-sectional survey

**DOI:** 10.1186/s44263-024-00080-3

**Published:** 2024-08-05

**Authors:** Chimwemwe Tembo, Linda Portsmouth, Sharyn Burns

**Affiliations:** 1 School of Population Health, Faculty of Health Sciences, Curtin University, Perth, Australia; 2 Saint John of God Hospitaller Services, Mzuzu, Malawi

**Keywords:** Perinatal anxiety, Maternal mental health, Adolescent mothers

## Abstract

**Background:**

The postnatal period is an important time for adolescent mothers to regain their health as they adjust to life with their infants. However, it is also a time when mothers are vulnerable to mental health problems. Generalized anxiety disorders (GADs) are among the common mental disorders that can impact mothers. Anxiety disorders can have adverse effects on a child’s cognitive development. However, there is a scarcity of studies pertaining to anxiety disorders among adolescent mothers in Malawi.

**Methods:**

A cross-sectional survey was conducted among adolescent postnatal mothers aged ≤ 19 years to establish the prevalence of probable GAD and identify cultural and social factors that influence anxiety in this population. Adolescent mothers were recruited from the Mitundu Rural Hospital catchment area in Lilongwe district, Malawi. A two-stage random sampling method was employed: clinics were randomly selected, and participants were recruited via systematic random sampling. The Generalized Anxiety Disorder (GAD-7) scale was used to assess anxiety. Data were analyzed using SPSS version 27.

**Results:**

Of the 395 adolescent mothers who participated, 34% were aged 14–17. The prevalence of probable GAD (*GAD-7* ≥ 10) was 31.9%. Increased social support decreased the odds of probable GAD (*aOR* 0.95, 95% *CI*: 0.91–0.98, *p* < 0.001). Experiencing intimate partner violence (IPV) increased the likelihood of probable GAD (*aOR* 4.80, 95% *CI*: 1.23–18.82, *p* = 0.02), while those who had contact with a health worker postnatally (*aOR* 0.38, 95% *CI*: 0.17–0.83, *p* = 0.02) and those who were “not prayerful” (*aOR*, 0.43, 95% *CI*: 0.21–0.87, *p* = 0.02) were less likely to report probable GAD.

**Conclusions:**

Given that the prevalence of probable GAD among adolescent mothers in Malawi is higher compared to the global estimates of their peers, policies and guidelines that prioritize the maternal mental health of adolescent mothers in Malawi are required. The findings also highlight the importance of enhancing social support among family and community. Co-designed mental health promotion, prevention, and early interventions to involve health workers and religious leaders are recommended.

## Background

Generalized anxiety disorder (GAD) is a common mental disorder associated with maladaptive parenting behaviors after childbirth [1]. Globally, the estimated prevalence of anxiety disorders during the first 12 months postnatal for women of all ages is 20% [2]. In low- and middle-income countries (LMIC), estimates range from 15 to 50% [3], compared with high-income countries (HIC), where the prevalence rate is estimated at 13.7% [3, 4]. Pregnancy and parenting during adolescence have been associated with poor mental health. Globally, anxiety disorders affect 50% of parenting teens [5]. However, about 70% of adolescent mothers with anxiety disorders are unrecognized and undiagnosed and do not receive any appropriate support [5, 6]. In Malawi, the prevalence of anxiety among perinatal adolescent women is unknown.

The presentation of anxiety symptoms during the postnatal period is similar to non-parenting women [7]. These symptoms include nervousness, worry, fear, irritability, tiredness, and fear of being alone [7]. In addition, anxiety may also manifest as physical problems such as sleep disturbance, physical tension, sweating, increased pulse rate, muscle tightness, body aches, and difficulty concentrating in response to ordinary life events [8]. In GAD, the symptoms of worry interfere with daily functioning [5, 9].

Similar to postpartum depression, untreated generalized anxiety disorder (GAD) has comparable impacts on the physical, mental, and social well-being of both the mother and the infant [3]. The impact on adolescent mothers can result in prolonged isolation from their peer group, a lack of financial resources, and poor coping strategies [5]. These issues may compromise mother-infant interaction with consequences associated with negative and disengaged parenting [3], overcontrolling maternal behaviors, neonatal neglect, failure to thrive, and infanticide [3]. In addition, anxiety disorders may result in longer-term health impacts for the mother and the child [3, 10]. For example, a longitudinal study in the United Kingdom (UK) found a significant association between maternal postnatal depression and anxiety of mothers and their child’s anxiety [11]. In the UK study, children of mothers who experienced postnatal anxiety were more likely to experience psychotic experiences in early adolescence [11].

The risk factors associated with maternal anxiety include the following: adverse childhood experiences (ACEs) such as violence, sexual abuse, and a family history of mental health disorders [9], a personal history of anxiety not related to the perinatal period, and contributing factors such as life stressors, poor relationship with the family, having a baby with health problems, social isolation, cultural factors, and poor social support [12]. However, specific risk factors for developing anxiety disorders have not been extensively researched [8, 11].

It is suggested cultural and ethnic factors influence anxiety disorders. This is considered to be due to differences in beliefs about the causes of illness [13] as well as cultural and social norms [14]. Culture plays an essential context in shaping the framework within which individuals perceive various phenomena, including experiences, common ideas, beliefs, attitudes, and norms, and affects how individuals experience mental health disorders [15]. The way perinatal mental health problems are understood and responded to by individuals is influenced by a person’s culture [15]. Cultural practices associated with childbirth may act as protective or risk factors for maternal mental health problems [16].

Compared to other sub-Saharan countries, Malawi has a higher number of adolescent mothers. Nevertheless, the existing body of research concerning anxiety disorders among adolescent mothers in Malawi is scarce, leaving a significant knowledge gap about the impact of social and cultural influences on anxiety in this specific population. This study describes findings from a broader study focusing on common mental disorders in adolescent mothers in Malawi [17, 18] and includes a specific focus on social and cultural factors associated with GAD during the postpartum period.

## Methods

### Study design

From 7 September 2021 through 31 March 2022, a cross-sectional survey was conducted among adolescent postnatal mothers aged 19 years or younger at maternal and child health (MCH) and outreach clinics at Mitundu Rural Hospital (MRH) as detailed in previous publications [17, 18]. A cross-sectional design was employed to provide a snapshot of self-reported mental health and cultural influences among adolescent mothers [19, 20].

### Setting

The study was conducted at Mitundu Rural Hospital (MRH) and its outreach clinics. The hospital is situated about 50 km from Lilongwe city [17, 18] and provides free maternal and child health (MCH) services, serving a rural population of 149,000 people. The adolescent fertility rate for women between the ages of 14–19 is 165 per 1000 women, with an average of 135 adolescent deliveries per month. Nearly half of all girls marry before the age of 18 [21]. The majority ethnic group in the area is the Chewa 43% [22]. Mitundu has 13 outreach child and maternal health clinics. The site was chosen because of the relatively high adolescent pregnancies, and it is a rural setting with a catchment area that is dominated by the Chewa ethnic tribe, who follow traditional cultural traditions surrounding childbirth, which include traditional ceremonies focusing on childbirth, family support and decision-making, traditional rules around cooking and socializing, and involvement of traditional healers and forced marriage [18].

### Study sample

To ensure a representative sample, a two-stage random sampling technique was utilized. In the initial stage, 7 of the 13 outreach clinics were selected using a simple random sampling method. During the second stage, systematic random sampling was employed, where mothers meeting the inclusion criteria were assigned a number from 1 to 3. Mothers allocated the number 3 were invited to participate, with approximately 59–60 mothers recruited across the 7 randomly selected sites. The study included postnatal adolescent mothers aged ≤ 19 who could provide informed consent [18]. To determine the appropriate sample size, the Slovin sample size determination formula was used: *n* = N/(1 + N*(e)2) [17], whereby *n* is the sample size, *N* refers to the population size, which was projected to be 30,653, according to the Lilongwe district report [23], and *e* is the level of precision (5% at 95% confidence level), resulting in a sample size of 395.

### Participants and public involvement

To ensure participation and public involvement, adolescent mothers were involved in the adaptation process of the data collection tool through a focus-group discussion. Partnerships with key stakeholders were also established to support the study. Additionally, an advisory committee consisting of hospital staff members, community volunteers, community health workers, community representatives, adolescent mothers, parent representatives, community health workers, and a member from Feed the Children International (a nongovernmental organization working in Mitundu) guided the study. The lead author organized regular meetings to discuss the study’s progress.

### Procedure for data collection

During data collection, clinic health workers and health surveillance assistants (HSAs) (community health field workers) introduced the researcher and trained research assistants (RA) to potential participants at each site. The aim of the study, the procedure involved, and the eligibility criteria were explained to mothers verbally in the Chichewa language. An information sheet in Chichewa was provided and read to interested eligible adolescent mothers by an RA. Ethical approval was granted for mature minor consent for participants under 18 years. Mature minor participants were asked additional questions to ensure they understood their potential involvement in the study. After obtaining verbal and written individual informed consent, the survey was administered verbally by trained RAs; administration took approximately 45 to 60 min. Data were recorded electronically using the Kobo toolbox; a secure digital questionnaire instrument software was used to collect and manage data for research [24].

### Data collection tool and measures

To assess levels of anxiety, the Generalized Anxiety Disorder-7 (GAD-7), a 7-item scale used for screening levels of anxiety experienced in the past 2 weeks, was used. The GAD-7 includes seven questions (response categories and scores range from *not at all* (0) to *nearly every day* (3)) and has been previously used in Malawi among adults [25]. In sub-Saharan Africa, Ghana, and Cote d’Ivoire, the GAD-7 demonstrated internal reliability of Cronbach alpha 0.68 coefficient, and the threshold score ≥ 10 reported a sensitivity of 89% and specificity of 80% for generalized anxiety [26]. For our study, the Cronbach alpha for GAD-7 items was 0.86. Research has supported the validity of GAD-7 as an interviewer-administered tool in local languages in Ghana, Malawi, and Cote d’Ivoire [25, 26]. For this study, a binary variable was created with a score of 0–9 as no/mild GAD and ≥ 10 as probable GAD, which is comparable with other studies [27]. Studies have indicated a score of 10 on GAD-7 can indicate the severity of worsening functionality and requires further evaluation [27]. Official guidelines for GAD-7 specify scores of 0–4, 5–9, 10–14, and 15–21 as cutoff points for screening for no, mild, moderate, and severe anxiety, respectively, with the recognition that the score does not provide a clinical diagnosis [26, 28]. For this study, the prevalence of no, mild, moderate, and severe anxiety has also been reported.

Demographics and social, behavioral, and cultural factors were collected. Demographics included age, number of births, tribe, occupation, education, marital status, type of marriage (polygamy or monogamy), and planned or unplanned pregnancy. Social factors were measured by the Multidimensional Scale of Perceived Social Support (MSPSS) and socioeconomic status. The MSPSS measures perceived social support from family, friends, or significant others [29]. Socioeconomic status was determined using the Wealth Index of the study population, which is a measure of household socioeconomic status in low- and middle-income (LMIC) countries based on the household ownership of consumer goods, dwelling characteristics, type of drinking water source, toilet facilities, and other characteristics [29, 30]. The assets included ownership of the following: land, animals (such as goats, cattle, chickens), radio, cellular phone, bed and/or mattress, sofa, table, chairs, refrigerator, bicycle, oxcart, motorcycle, roofing materials, and flooring materials use of electricity, lamps, radio, television, and water source. The Wealth Index was compared within households in rural settings. Behavioral questions included tobacco and alcohol use and experience of intimate partner violence (IPV). IPV was measured using the Hurt, Insult, Threaten, and Scream (HITS) scale. HITS comprised a 5-point Likert scale; responses included never to frequently (scores range 0–25) [31]. Scores of ≥ 10 determined IPV experiences [31]. Cultural factors included questions about common cultural practices, such as support from religious and traditional leaders, family and community support, and community norms (see Table 1). Responses were binary (“yes” or “no”). Questions about common cultural factors were generated from the literature, and the content was validated as part of the survey validation which has been described elsewhere [17, 18].

**Table 1:** Cultural characteristics of participants

Cultural factors	Total *n* = 395 *N* (%)	No GAD *N* (%)	Probable GAD *N* (%)	*χ*^2^ statistic	*p*-value
**Seek help from religious for mental health issues**				2.268	0.13
Yes	216 (55)	152 (70.4)	64 (29.6)		
No	179 (45)	138 (77)	41 (22.9)		
**Family freedom to discuss mental health**				20.213	< 0.001***
Yes	187 (47)	157 (84)	30 (16.1)		
No	208 (53)	133 (63.3)	75 (36.1)		
**Negative reaction to pregnancy by family and relatives**				4.587	0.032*
Yes	150 (38)	101 (67.3)	49 (32.7)		
No	245 (62)	189 (77.1)	56 (22.9)		
**Being isolated in family activities because of pregnancy**					
Yes	47 (11)	23 (48.9)	24 (51.1)	16.383	< 0.001***
No	348 (89)	267 (76.7)	81 (26.6)		
**Family positive about gender of the child**				0.240	0.68
Yes	304 (77)	225 (74)	79 (26)		
No	91 (23)	65 (71.4)	26 (28.6)		
**Family making decisions on your behalf regarding your care**					< 0.001***
Yes	304 (77)	238 (78.3)	66 (21.7)	16.047	
No	91 (23)	52 (57.1)	39 (4.2)		
**Strict cultural rules, beliefs, and practices regarding pregnancy and childbirth**				1.867	0.18
Yes	342 (87)	247 (72.2)	95 (27.8)		
No	53 (13)	43 (81.6)	10 (18.9)		
**Lack interaction with the health care providers**				19.478	< 0.001***
Yes	66 (17)	34 (51.5)	32 (48.5)		
No	329 (83)	256 (77.8)	73 (22.2)		
**Forced to get married to someone**				1.393	0.23
Yes	114 (29)	79 (69.3)	35 (30.7)		
No	281 (71)	211 (75.1)	70 (24.9)		
**Being supported by husband**				13.059	< 0.001***
Yes	293 (74)	229 (78.2)	64 (21.8)		
No	102 (26)	61 (59.8)	41 (40.2)		
**You have become more prayerful to seek God for help**					
Yes	125 (32)	72 (57.6)	53 (42.4)	23.444	< 0.001***
No	270 (68)	218 (80.7)	52 (19.3)		
**Being forced to have sex with a man because of cultural practices**				5.208	0.022*
Yes	44 (11)	26 (59.1)	18 (40.9)		
No	351 (89)	264 (75.2)	87 (24.8)		
**Experiencing restrictions to cook and socialize because of pregnancy & delivery**				2.208	0.137
Yes	220 (55.6)	168 (76)	52 (24)		
No	175 (44.4)	122 (68)	53 (30)		
**Information regarding childbirth and parenting from community elders**				0.011	0.91
Yes	326 (83)	239 (74)	87 (26)		
No	69 (17)	51 (74)	18 (26)		

^*^Statistically significant at *p* < 0.05 and ***statistically significant at < 0.001

### Translation of survey questionnaire

All questions and scales, including GAD-7 and HITS, were translated using the World Health Organization’s standardized and validated translation process [32]. Firstly, questions were translated from English to the local language, Chichewa, and then back to English by an independent professional translator. Two senior researchers and two mental health workers assessed construct and content validity. Secondly, using the translated questionnaire, one focus-group discussion with 15 adolescent mothers was conducted to ensure face validity [33]. Finally, vague or ambiguous words and phrases were discussed and agreed upon. Due to reading difficulties among many adolescent mothers, all questions were asked orally in the Chichewa language. The lead author (C. T.) trained two RAs with mental health professional backgrounds to ensure standardized questionnaire administration.

### Data analysis

Data were collected electronically through Kobo Toolbox and then exported to the Statistical Package for Social Sciences (SPSS) version 27 software for analysis. The dependent variable of interest was generalized anxiety disorder (GAD), which was computed into a binary outcome (GAD-7 scores < 10 no/mild GAD and scores ≥ 10 as probable GAD). Independent variables of interest include all demographic, social, behavioral, and cultural variables. Socio-economic status was computed as a Wealth Index using the principal component analysis (PCA) methodology. The Wealth Index was calculated by combining variables of asset ownership and housing characteristics within the population under study into the proxy indicator Wealth Index. Firstly, frequencies were computed by selecting each asset and exploring the frequencies of each asset variable. If an asset was owned by less than 5% and more than 95% of the households, it was excluded. Then, scores were generated using PCA methodology for the Wealth Index, which was recorded into five quintiles, with the 1st the poorest and the 5th the wealthiest. Descriptive statistics of demographic, behavioral, and cultural factors were tabulated. To calculate the prevalence of GAD, descriptive analysis using the frequency function was performed on the recorded binary GAD variable. A bivariate analysis using the Pearson chi-square test of association for categorical response variables and an independent *t*-test was then performed between the dependent variable GAD and MPSS scores. Chi-square was tabulated with each of the categorical independent variables to determine association. Covariates in the model included all significant factors identified via the chi-square test of association and the *t*-test: age, marital status, polygamy, age at marriage, occupation, place of delivery, level of education of spouse, MSPSS, Wealth Index, family freedom, being isolated, interaction with a health worker, forced marriage, husband support, being prayerful, experience of loss, and IPV. Odds ratios, confidence intervals, coefficients, and *p*-values were obtained. Statistical significance was based on a two-sided test at *p* < 0.05.

## Results

### Demographic characteristics and association with probable GAD

Of the 401 participants who were approached, 395 were interviewed resulting in a response rate of 99.5%. The prevalence of probable GAD was 31.9% (*n* = 126) (Table 2). Seventy-eight percent (*n* = 308) of participants were married; 34% (*n* = 113) were married before they became pregnant. Sixty-four percent (*n* = 253) reported an unplanned pregnancy. Of the married participants, 12.3% (*n* = 38) were in a polygamous marriage. Most participants identified as Christians 95.7% (*n* = 378). No education or only primary education was reported by 88% (*n* = 355) of participants (Table 3). Single participants reported a higher prevalence of probable GAD (*p* < 0.001) when compared to married participants. Participants in a polygamous relationship exhibited a higher likelihood of presenting probable GAD (*p* = 0.047) compared to those in a monogamous relationship. There was no significant association between the level of education and probable GAD (*p* = 0.49); however, participants with a spouse who had low education were more likely to report probable GAD compared to those whose spouses had a higher education (*p* < 0.001). Participant age, age at marriage, level of education, gender of the child, place of delivery, planned or unplanned pregnancy for this child, and history of anxiety did not show a significant association with probable GAD in this sample.

**Table 2:** Prevalence of generalized anxiety

GAD severity	Frequency	Percentage
No anxiety (0–4)	111	28.1%
Mild anxiety (scores 5–9)	158	40.0%
Moderate anxiety (scores 10–14)	85	21.5%
Severe anxiety (15 and above)	41	10.4%

Generalized anxiety disorder (GAD-7) [34]

**Table 3:** Demographic characteristics of participants

Variable	Total *n* = 395 *N* (%)	No/mild GAD *N* (%)	Probable GAD N (%)	*χ*^2^ statistic	*p*-value
**Demographic factors**					
**Age (years)**					
13–17	135 (34.1)	95 (71.4)	38 (28.6)	0.611	0.434
18–19	260 (65.9)	193 (75.1)	64 (24.9)		
**Marital status**					
Single	65 (16.5)	40 (61.5)	25 (38.5)	21.192	0.001***
Married	308 (78.0)	241 (78.2)	67 (21.8)		
Divorced/widowed	22 (5.5)	9 (41)	13 (59)		
**Polygamy marriage**				3.952	0.047*
Yes	38 (12)	25 (65.8)	13 (34.2)		
No	270 (88)	216 (80)	54 (20)		
**Age at marriage**					
13–17	226 (74.6)	172 (76.1)	54 (23.9)	3.307	0.069
18 and over	169 (25.7)	158 (85.9)	11 (14.1)		
**Occupation**				13.215	0.021*
Student	17 (4.3)	9 (52.9)	8 (47.1)		
Farmer	366 (93)	272 (74)	94 (26)		
Business	12(4)	11 (91.7)	(8.3)		
**Level of education**				1.410	0.49
Never	5 (1.3)	4 (80)	1 (20)		
Primary	350 (88.6)	259 (74)	91 (26)		
Secondary	40 (10.1)	27 (67.5)	13 (32.5)		
**Level of education of the spouse**				9.026a	0.001***
None	22 (100)	22 (100)	0 ()		
Primary	229 (58)	171 (74.7)	58 (25.3)		
Secondary	57 (14)	48 (84.2)	9 (15.8)		
**Gender of the baby**				0.358	0.56
Male	182 (46)	131 (72.6)	51 (28)		
Female	213 (54)	159 (74.6)	54 (25.4)		
**Planned or unplanned Pregnancy**				1.726	0.63
Yes	145 (38)	112 (77.2)	33 (22.8)		
No	250 (62)	178 (71.2)	72 (28.8)		
**Place of delivery**				1.726	0.63
Hospital	366 (93)	271 (74.0)	95 (26.0)		
Traditional birth attendants	29 (7)	19 (65.5)	10 (34.5)		
**Religion**				1.372	0.84
Christian	378 (95.7)	275 (73)	103 (27)		
Other religions	17 (4.3)	14 (82)	3 (18)		
**Ethnic group**				2.751	0.43
Chewa	387 (98)	283 (73.1)	104 (26.9)		
Other	8 (20)	7 (87.5)	1 (12.5)		
**Family history of a mental disorders**					
Yes	16 (4)	12 (75)	4 (25)	0.063	0.96
No	358 (90)	263 (73.5)	95 (26.5)		
Unsure	21 (5.3)	15 (71.4)	6 (28.6)		

^***^Statistically significant at *p* < 0.001 and *statistically significant at < 0.05

### Behavioral characteristics and association with probable GAD

Participants were relatively evenly spread across the five quintiles of the Wealth Index (see Table 4). IPV was experienced by 5.3% (*n* = 21) of participants. Participants who had experienced IPV were more likely to report probable GAD (*p* < 0.001) than those who had not experienced IPV. Those from the poorest (1st) and wealthiest quintile (5th) were more likely to report probable GAD (*p* < 0.001) compared to those from the 2nd and 3rd quintiles. Consumption of alcohol was reported by only 2% (*n* = 8) of participants, and no participant reported smoking tobacco.

**Table 4:** Social and behavioral characteristics and association with probable GAD

Variable	Total *n* = 395	No/mild GAD *N* = 269 (68.1%)	Probable GAD *N* = 126 (31.8%)	*χ*^2^ statistic	*p*-value
**Behavioral characteristics**					
**Current alcohol consumption**					0.21
Yes	8 (2)	4 (50)	4 (50)		
No	387 (98)	383 (97)	4 (1.3)		
**Intimate partner violence**					
No	374 (94.7)	283 (75.7)	91 (24.3)	18.260	< 0.001***
Yes	21 (5.3)	7 (33.3)	14 (66.7)		
**Social characteristics**					
**MPSS (*t*-test)**	7.034				< 0.001***
**Wealth index**					
1st quintile	74 (19)	11 (14.9)	63 (85.1)	23.743a	< 0.001***
2nd quintile	106 (27)	45 (42.5)	61 (57.5)		
3rd quintile	55 (14)	10 (18.2)	45 (81.8)		
4th quintile	79 (20)	22 (27.8)	57 (72.2)		
5th quintile	76 (20)	14 (18.4)	62 (81.6)		

^*^Statistically significant at *p* < 0.05 and ***statistically significant at < 0.001

### Cultural factors and association with probable GAD

Many participants reported to have sought help from religious leaders to treat mental health problems (*n* = 212, 54.4%). More than half (*n* = 206; 52.8%) reported they did not have family freedom to discuss mental health issues. Over one-third of mothers (*n* = 150; 38%) had experienced negative reactions to their pregnancy by family and relatives. Family and relatives’ reaction towards the gender of their baby was generally positive (*n* = 304; 77%). Forty-four percent (*n* = 172) of participants experienced restrictions to cooking and socializing because of their pregnancy and delivery. Sixteen percent (*n* = 66) experienced a lack of interaction with health care providers, and 86.4% (*n* = 337) reported they had to follow cultural rules strictly after childbirth. Participants who perceived that they had no freedom to discuss issues within their family were more likely to report probable GAD (*p* < 0.001) compared to those who were able to discuss mental health with their family. In families where decisions were not made on behalf of the adolescent mothers, participants were more likely to report probable GAD (*p* < 0.001). Participants who reported receiving poor support from their partner (*p* < 0.001) were more likely to report probable GAD (*p* < 0.001) compared to those who had supportive spouses. Additionally, participants who lacked interaction with healthcare providers had a higher likelihood of reporting probable GAD than those who had interacted with health workers (*p* < 0.001).

Table 5 describes responses for the seven GAD symptoms experienced during the last 2 weeks. “Worrying too much about different things’ for several days and more was the most common symptom reported (74.9%; *n* = 296), followed by ‘feeling afraid that something awful might happen’ for several days or more (71.6%, *n* = 283) and ‘being irritable’ for several days or more (70.7%, *n* = 279).”

**Table 5:** GAD reported symptoms experienced during the last 2 weeks

Variable	Not at all	Several days	More than half the days	Nearly everyday	Mean	Standard deviation
Feeling nervous, anxious, or on edge	145 (36.7%)	154 (39%)	65 (16.5%)	31 (7.8%)	0.95	0.92
Not being able to stop or control worrying	120 (30.4%)	171 (43.3%)	73 (18.5%)	31 (7.8%)	1.04	0.896
Worrying too much about different things	99 (25.1%)	154 (39. 0%)	91 (23%)	51 (12.9%)	1.24	0.971
Trouble relaxing	132 (33.4%)	150 (38%)	64 (16.2%)	49 (12.2%)	1.08	0.995
Being so restless that it is hard to sit still	179 (45.3%)	132 (33.4%)	45 (11.4%)	39 (9.9%)	0.86	0.972
Becoming easily annoyed or irritable	116 (29.4%)	154 (39%)	77 (19.5%)	48 (12.2%)	1.14	0.98
Feeling afraid as if something awful might happen	112 (28.4%)	145 (36.7%)	74 (18.7%)	64 (16.2%)	1.23	1.034

After the bivariate analysis was conducted, significant covariates were checked for multicollinearity before inclusion in the logistic regression model. The multicollinearity test revealed no multicollinearity among the independent variables with a variance inflation factor (VIF) of less than 10 for all variables except for socioeconomic status. Therefore, socioeconomic status was excluded from the model. A binary logistics regression model found an inverse relationship between MSPSS and GAD, whereby increased support decreased the odds of probable GAD occurring (adjusted odds ratio (aOR) 0.95, 95% *CI*: 0.91–0.98, *p* < 0.001) (Table 6). Participants who had experienced IPV had increased odds of reporting GAD symptoms (*aOR* 4.80, 95% *CI*: 1.23–18.82, *p* = 0.02). Those who had contact with a health worker postnatally were less likely to report symptoms than those who lacked interaction with a health worker (*aOR* 0.38, 95% *CI*: 0.18–0.84, *p* = 0.02). Participants who reported not being more prayerful were less likely to present GAD symptoms (*aOR*, 0.04, 95% *CI*: 0.22–0.87, *p* = 0.02) than those who reported to be more prayerful.

**Table 6:** Factors associated with high levels of GAD

Variable	Odds ratio	*p*-value	B coefficient	Confidence interval
Multidimensional social support	0.95	< 0.001***	− 0.055	0.91–0.98
**Intimate partner violence**				
No	Reference			
Yes	4.80	0.02*	1.570	1.23–18.81
**Interaction with healthcare providers**				
No	Reference			
Yes	0. 38	0.016*	− 0.955	0.18–0.83
**Being prayerful**				
Prayerful	Reference			
Not prayerful	0.43	0.02*	− 0.976	0.22–0.87

^*^Statistically significant at *p* < 0.05.^***^Significance at < 0.001. CI calculated at 95% (significant at *p* < 0.05). Reference group. Adjusted for all variables in the table as well as age, experiencing loss, marital status, polygamy, spouse education, family, freedom, negative reactions, feeling isolated, family decisions, not being supported, and forced sex

## Discussion

This is the first study to assess anxiety among postnatal adolescent mothers in Malawi. The prevalence rate (31.9%) is higher than global estimates of self-reported anxiety among postnatal women (17.8%) [3] and higher than a UK study among adolescent mothers aged 15–19, which reported a prevalence of 16.7% [35]. Despite differences in prevalence rates and the use of different screening tools, there is evidence that supports the claim that 15–19-year-old mothers report higher rates of anxiety compared to adult mothers [11, 35]. The prevalence of anxiety may vary across countries due to diversity in settings, recruitment strategies, data collection strategies, and measurement tools, the complexity of anxiety, different types of anxiety, and cultural influences [36]. The GAD-7 tool used in this study has a comparative efficacy with the Edinburgh Postnatal Depression Scale (EPDS), which comprises emotional and cognitive symptoms of anxiety or depression [37]. In this population, most social and cultural factors that predicted probable postnatal depression using EPDS [18] also predicted probable anxiety using GAD-7. There were, however, exceptions for the some social and cultural factors, for example, “family making decision on adolescent mother’s care” predicted probable postnatal depression when using EPDS [18]. However, this cultural factor did not predict probable GAD. Spouse education level and being forced to have sex with a man were associated with anxiety but not with probable postnatal depression [18].

Consistent with studies in Brazil [38], South Africa [39], and Portugal [40], IPV was found to be a predictor of GAD among perinatal young women. However, the rates of IPV in this study were lower than those reported in the United States (US), which found a prevalence of 17.5–25% among adolescent mothers of different ethnicities. The US study found many adolescent mothers experienced IPV for the first time in their postnatal period [41]. In this study, low rates of IPV may be attributed to underreporting by adolescent mothers who may fear their partners will respond with harsh punishment if they disclose. Nevertheless, it is imperative that systematic screening for IPV and anxiety be enhanced in practice, especially at each point of contact with adolescent mothers to enhance early identification and support mothers with appropriate interventions [40].

Health care provider interaction was found to be a protective factor for GAD in this study. Early identification of GAD by health workers was found to prevent worsening of GAD symptoms among perinatal women in Portugal [40]. Our findings suggest that increased trust by women in their healthcare professionals may foster more open and honest communication about psychological distress.

Being not prayerful was associated with a lower likelihood of reporting probable GAD compared to being more prayerful. A study of adolescent mothers in Indiana found maternal participation in religion, and prayerfulness were used as coping mechanisms when exposed to traumatic life events [42]. This may suggest adolescent mothers experiencing stressors may look for a new sense of purpose in their lives and seek religion and spirituality [43]. In contrast to our study, a study with 248 pregnant and postnatal mothers in California found that being prayerful or religious was associated with better mental health [44]. In Malawi, religiosity is considered a central aspect of culture; therefore, life events are interpreted in religious terms, and prayer and religion offer unique coping pathways to stress. It may be that participants in this study experiencing stressors were more likely to seek religion and prayerfulness. These findings highlight the need to engage religious leaders and design mental health interventions with a spiritual or pastoral care focus.

Social support was found to be a protective factor among adolescent mothers. Similarly, studies in Turkey [45] and Brazil [46] found social support appeared to reduce the risk of GAD among adolescent mothers. Social support is believed to play a significant role in enhancing an individual’s capacity to cope with stressors [44, 47]. Increased support served as a protective factor for adolescent mothers in this study, with findings indicating that some predictors of GAD for adolescent mothers are socially and culturally oriented. The Malawian context is communal rather than individualistic; hence, societal support is essential for mental health. Consistent with Bronfenbrenner’s (1994) bioecological model of human development [48] and the influence of social and cultural factors found in this study, family and community are important to consider when developing mental health prevention and promotion interventions for adolescent mothers.

This study found that level of education, if the pregnancy was planned or unplanned, and history of mental health disorders were not significantly associated with probable GAD. These results are inconsistent with a South African study among adult perinatal women that found mental health disorders, unplanned pregnancy, and food insecurity to be associated with increased risk of anxiety [49]. A study of Israeli adult women found those with higher education status were more likely to suffer postpartum anxiety [4]. However, a meta-analytic synthesis of risk factors of new onset anxiety in the perinatal period (*n* = 961) found a low level of education attainment to be a risk factor for perinatal anxiety, and higher education attainment was not a significant influencing factor [50].

While this study identified several social and cultural factors that were associated with probable GAD in adolescent mothers, there are few other studies that have explored demographic, social, behavioral, and cultural factors and the association with GAD among adolescent mothers in LMIC to compare with these results. However, the findings challenge the idea that women’s mental health during the postnatal period is protected by culturally prescribed traditional postpartum rituals [51, 52].

The findings of this study highlight the need for prevention and promotion strategies to increase awareness of and enhance help seeking for maternal mental health issues among adolescent mothers. There is a need to consider other community settings and the role of religious leaders in the implementation of strategies to promote good maternal mental health. The findings also call for the inclusion of maternal mental health interventions for postpartum mothers within existing policies and guidelines, with a focus on the promotion of the mental health of adolescent mothers in the postnatal period beyond the routine care that is currently provided. Routine screening for adolescent mothers to identify the risk of anxiety disorders and other issues such as IPV is needed.

There are some limitations to this study. As this study is cross-sectional, causal pathways cannot be assumed; however, potential predictors are identified and can inform health promotion strategies and interventions. The cross-sectional design may also provide information to generate hypotheses that inform other future study designs [19]. Participants were recruited from clinics using a systematic random sampling method. Given the population group, it was deemed most appropriate to recruit mothers when they attended the clinic for their baby checkups.

## Conclusions

This study found social support to be a protective factor against probable GAD among adolescent mothers, highlighting the need to consider cultural and social networks within the support system. Considering the importance of social support, family, community, and adolescent mothers’ involvement in the co-design of mental health prevention and promotion interventions for adolescent mothers in Malawi, is essential. Interaction with health care workers was also found to be protective against anxiety, reinforcing the clear policies and procedures, including adequate staff as well as mandatory screening for anxiety and other common mental disorders, to ensure pre- and postnatal adolescent mothers receive appropriate prevention and early intervention.

## Data Availability

The Curtin University Survey Office, which manages the Human Research Ethics Committee for researchers, allows limited access to data generated for this study to protect participants’ privacy and related confidentiality agreements. However, interested researchers may reach out to the Human Research Ethics Committee (hre@curtin.edu.au), or the chairperson of the Human Research Ethics Committee and the corresponding author, Mrs. Chimwemwe Tembo (chimweptembo@yahoo.co.uk), to request access to data if needed to reproduce the article or review results.
